# Supplementary material for: Weakly-supervised explainable infection severity classification from chest CT scans
Source: PLoS One. 2025 Oct 30;20(10):e0334431. doi: 10.1371/journal.pone.0334431 (PMC12574926; doi:10.1371/journal.pone.0334431)
Supplement: Attachment — Submitted filename: Response to Reviewers.pdf [file pone.0334431.s001.pdf]

We thank the reviewers for their efforts in reviewing this work and for their constructive feedback. In this document, we address each point raised by the reviewers, providing comments and referring them to changes we have made in the revised manuscript.

Reviewer #1: The authors present a weakly-supervised machine learning system to classify COVID-19 infection severity from chest CT scans. The paper is very well written, all steps of the classification pipeline are fully understandable. I thus have no comments for improvement.

Response: We thank Reviewer #1 for their comments about our paper.

Reviewer #2: This paper presents a weakly supervised classification method for how to categorize infection severity from chest CT scan images and provide clinicians with interpretable results.

1. Weakly supervised classification method:

The paper proposes a weakly supervised training method to train a Cuboid Nominator by combining the outputs of volume-level and cuboid-level classifiers. This approach avoids the strong reliance on labeled data and significantly reduces the need for manual labeling by training through pseudo-labeling and self-supervised learning.

2. Cuboid Nominator:

A 3D CNN-based cuboid nominator model is proposed, which is able to select “useful” cuboids from the input CT volume data. By extracting meaningful local regions from the overall volume data, this method focuses more on the extraction of local features than traditional methods, which helps to improve the accuracy of classification.

3. Cube-level classification:

The paper further proposes a small 3D CNN model specifically for classifying nominated cubes.By inferring whether each cube is an infected region through pseudo-labeling, this refined classification strategy helps to extract more precise features and improve the overall classification.

4. Introduction of Infection Clusters:

The paper proposes that lung infections are usually manifested as a cluster of neighboring cubes, and therefore a mechanism is devised such that the classification of each cube not only depends on itself, but also interacts with the features of the surrounding cubes. This approach takes into account the spatial correlation of the infection region and can effectively improve the classification performance, especially when dealing with data with spatial dependencies.

5. Volume-level classifier::

A volume-level classifier based on a multilayer perceptual machine (MLP) is introduced, which can better understand the spatial relationship between cubes by combining the representation of each cube and its positional encoding, thus improving the accuracy of classification.

The shortcoming include:

1. Higher model complexity:

The method introduces multiple model components (cube nominator, cube classifier, volume level classifier, etc.), and although the design of these components helps to improve the model performance, it also brings higher computational cost and model complexity. A large amount of training data and computational resources are required, which may be difficult to implement in some resource-limited environments.

2. Limitations of the infection cluster model:

The assumption for the construction of infection clusters is based on the assumption that infected regions are spatially correlated, but this assumption is not always applicable to all types of infections, especially since some infections may exhibit a non-clustered morphology. Therefore, this approach may not be applicable in some special cases.

3. Dataset limitations:

The paper mainly used two datasets, and although these datasets are from different countries and hospitals, there may still be geographical and clinical practice differences, which may affect the model's ability to generalize.

These shortcomings provide directions for future research, helping to enhance the model's practicality and effectiveness.

References：

Ji Z, Mu J, Liu J, et al. ASD-Net: a novel U-Net based asymmetric spatial-channel convolution network for precise kidney and kidney tumor image segmentation[J]. Medical &

Biological Engineering & Computing, 2024: 1-15. https://doi.org/10.1007/s11517-024-03025-y

Ji Z, Zhao J, Liu J, et al. ELCT-YOLO: an efficient one-stage model for automatic lung tumor detection based on CT images[J]. Mathematics, 2023, 11(10): 2344. https://doi.org/10.3390/math11102344

* Your results need to be compared with two papers, and the analysis can be conducted in the Introduction section.

Response: We thank **Reviewer #2** for their comments and constructive feedback. We address each of the shortcomings put forward by the reviewer as follows:

1. Higher model complexity: We describe our method's minimal added computational complexity in the Experiments section of our paper. It is worth noting that the added computational overhead from the cuboid and volume classifiers is very low. The Densenet-121 requires 344.121 GFLOPs at inference time, while our region proposal model requires 344.572 GFLOPs. The volume classifier is a simple MLP that carries out the classification based on the latent from the cuboid nominator and classifier, while the cuboid classifier takes in the nominated cuboid (16x64x64) to classify it.
2. Limitations of the infection cluster model: In the Infection Clusters subsection, we discuss in more specific terms the types of infections for which such a model will be effective. The infection cluster assumption would be effective for other clustered infections, such as Lobar Pneumonia, Bronchopneumonia, Interstitial Pneumonia, and Pleural Effusion. However, it would be unhelpful for an infection like Miliary Pneumonia, which presents in small pulmonary nodules scattered across the lungs.
3. Dataset Limitations: The primary motivation of using datasets from different countries and hospitals was to ensure the model's ability to generalize across diverse populations, imaging protocols, and clinical settings. While geographical and clinical practice differences exist, incorporating multiple datasets with varied characteristics strengthens the model's robustness rather than limiting its generalizability. By exposing the model to heterogeneous data distributions, it learns to handle variations in radiographic techniques, patient demographics, and disease presentations. Moreover, evaluating the model on multiple independent datasets provides a more rigorous assessment of its real-world applicability, reducing the likelihood of overfitting to a single institution’s imaging patterns. To further address potential biases, we could in the future explore additional external validation or domain adaptation techniques.

4. Additional References: It is difficult to compare directly with Segmentation models as the datasets we use do not have ground-truth Segmentation masks. However, we discuss them in the introduction section (Explainability) as they are still relevant.

Reviewer #3: 1. Authors are required to clearly highlight how the proposed weakly-supervised approach contributes to clinical adoption.
2. The strategies used to handle class imbalance should be explained more with clarity.
3. Expand the comparison with other methods beyond the F1 score and accuracy metrics.
4. Include additional insights into the model's generalizability by evaluating it on other respiratory conditions, as the study currently focuses on COVID-19.
5. Provide a more detailed explanation of critical steps, such as the "cuboid nomination" process and its role in infection localization.
6. Expand the discussion on limitations and future work, particularly the potential of integrating multimodal data.
7. Improve the clarity of visualizations especially fig 5 and 7.
8. Revise conclusion by highlighting the key take aways from the paper.

Response: We thank **Reviewer #3** for their comments and constructive feedback. We address each of their requirements as follows:

1. We agree that clinical adoption is an important point to show the clinical relevance of this work. To this end, we highlight this work's contribution to clinical adoption in the Conclusion and Discussion sections.
2. In the experiments section, we add details about the stratified sampling employed to deal with class imbalance. This is mentioned together with the weighted cross entropy description.
3. We have also reported precision and recall compared with other methods. In the Results section, we discuss those comparisons further.
4. We agree with the reviewer that checking the model's generalizability to other respiratory conditions is important. However, we have used this as a proof-of-concept case study due to the limited availability of CT lung infection data beyond COVID-19.
5. In the methodology section, we provide more details surrounding the "cuboid nomination," highlighting its role in infection localization.
6. We address the limitations and future work further in the conclusion section.
7. We improved the resolution of Figures 5 and 7. However, it is worth noting that sometimes, during the review process, figures are included in lower resolution.
8. We revise the conclusion while also addressing points 1 and 6.
