# Supplementary material for: Weakly-supervised explainable infection severity classification from chest CT scans
Source: PLoS One. 2025 Oct 30;20(10):e0334431. doi: 10.1371/journal.pone.0334431 (PMC12574926; doi:10.1371/journal.pone.0334431)
Supplement: Attachment — Submitted filename: Response_to_Reviewers_auresp_2.pdf [file pone.0334431.s002.pdf]

We thank the reviewers and academic editor for their efforts in reviewing this work and for their constructive feedback. In this document, we address each point raised by the reviewers, providing comments and referring them to changes we have made in the revised manuscript.

Reviewer #3: The method is technically sound and shows strong performance across datasets.

Minor suggestions:

1, Consider briefly discussing how this method could fit into clinical workflows.

2, Visuals like Fig. 5 and Fig. 7 could still benefit from slight clarity improvements.

Overall, a solid paper just a few small refinements needed.

Response: We thank the Reviewer for their comments about our paper. Below are our responses to their minor suggestions:

1. To better highlight the potential impact of our work in clinical workflows, we have added a dedicated paragraph (Page 13, Line 462) in the conclusion to discuss this. This addition clarifies how our work contributes to clinical practice and future research directions.

2. We improved the clarity of the figures in the paper, particularly figures 5 and 7.
