# Supplementary material for: Weakly-supervised explainable infection severity classification from chest CT scans
Source: PLoS One. 2025 Oct 30;20(10):e0334431. doi: 10.1371/journal.pone.0334431 (PMC12574926; doi:10.1371/journal.pone.0334431)
Supplement: Attachment — Submitted filename: Response_to_Reviewers_auresp_3.pdf [file pone.0334431.s003.pdf]

We thank the reviewers and academic editor for their efforts in reviewing this work and for their constructive feedback. In this document, we address each point raised by the reviewers, providing comments and referring them to changes we have made in the revised manuscript.

Reviewer #3: the paper is very well written, I have minor recommendations that would improve the paper quality much better.
Please provide a bit more detail on your model architecture and training settings to help with reproducibility also
Consider elaborating on the limitations and possible real world challenges of your approach and the last one to add few figures could be improved for clarity.

Response: We thank the Reviewer for their comments about our paper. Below are our responses to their minor suggestions:

1. We have added the Github repo to the code implementation and the hyperparamteres used to achieve each metric. We have provided the link in the paper in footnote of page 3.

2. We added an extension to the limitation description in the conclusion that describes the impact of using the MosMed dataset subset on the explainability validation. As for the clarity of the figures, we recomment to view the the full-resolution figures using the download links at the top right hand corner of the figure pages.
